# Regional variation in hospital care at the end-of-life of Dutch patients with lung cancer exists and is not correlated with primary and long-term care

**DOI:** 10.1093/intqhc/mzaa004

**Published:** 2020-03-18

**Authors:** Yvonne de Man, Stef Groenewoud, Mariska G Oosterveld-Vlug, Linda Brom, Bregje D Onwuteaka-Philipsen, Gert P Westert, Femke Atsma

**Affiliations:** 1 Radboud University Medical Center, Radboud Institute for Health Sciences, IQ Healthcare, Nijmegen, The Netherlands; 2 Department of Public and Occupational Health, Amsterdam Public Health Research Institute, Expertise Center for Palliative Care, Amsterdam UMC, Vrije Universiteit Amsterdam, Amsterdam, The Netherlands, Van der Boechorststraat 7, 1081, BT, Amsterdam, The Netherlands, and; 3 IKNL, Netherlands Comprehensive Cancer Organization, Godebaldkwartier 419, 3511 DT Utrecht, the Netherlands

**Keywords:** practice variations, health policy, quality improvement, hospital medicine, primary care, health services research, cancer

## Abstract

**Objective:**

To examine the regional variation in hospital care utilization in the last 6 months of life of Dutch patients with lung cancer and to test whether higher degrees of hospital utilization coincide with less general practitioner (GP) and long-term care use.

**Design:**

Cross-sectional claims data study.

**Setting:**

The Netherlands.

**Participants:**

Patients deceased in 2013–2015 with lung cancer (*N* = 25 553).

**Main Outcome Measures:**

We calculated regional medical practice variation scores, adjusted for age, gender and socioeconomic status, for radiotherapy, chemotherapy, CT-scans, emergency room contacts and hospital admission days during the last 6 months of life; Spearman Rank correlation coefficients measured the association between the adjusted regional medical practice variation scores for hospital admissions and ER contacts and GP and long-term care utilization.

**Results:**

The utilization of hospital services in high-using regions is 2.3–3.6 times higher than in low-using regions. The variation was highest in 2015 and lowest in 2013. For all 3 years, hospital care was not significantly correlated with out-of-hospital care at a regional level.

**Conclusions:**

Hospital care utilization during the last 6 months of life of patients with lung cancer shows regional medical practice variation over the course of multiple years and seems to increase. Higher healthcare utilization in hospitals does not seem to be associated with less intensive GP and long-term care. In-depth research is needed to explore the causes of the variation and its relation to quality of care provided at the level of daily practice.

## Introduction

Often, care for patients near the End-of-Life (EOL) does not meet the desires of patients or their families [1] and pro-active palliative care is not provided on time [2]. Increasing signals of potential inappropriate care and healthcare costs at the EOL have led to the research of possible overuse as well as underuse of many medical procedures [3–6].

For patients with advanced illness, and specifically for cancer patients, there can be under-treatment of pain and other burdensome symptoms, as well as over-treatment with curative therapies that many patients might not want [7]. Particularly of interest in the EOL phase are aggressive treatments. For instance, the Dartmouth Atlas reports that many patients in the terminal stages of cancer undergo chemotherapy that does not always lengthen life but all too often makes their last days more miserable [8].

Lung cancer (LC) is a common cancer type with high hospital care costs, and patients are very likely to undergo aggressive treatments [9]. They are heavy users of acute care beds and the emergency room (ER) at the EOL [9,10], both indicators of poor-quality care [10]. In a previous study, we already described hospital care utilization at the EOL and concluded that the percentage of patients with LC with EOL hospital admissions, ER contacts and CT scans was high (66%, 57% and 65%), pointing to potential overuse of medical care. In contrast, utilization of palliative consultations was low (3%) [-Reference blinded for review], even though it is known that early palliative care can optimize the quality of EOL care [11,12] and the treatment decision ‘potentially curative/life-prolonging treatment’ was more often described as inappropriate (35%) than appropriate (8%) [13].

Examining clinical variation in medical practice is an important first step to measuring efficiency and effectiveness in care delivery [14]. Variation in healthcare utilization can raise questions regarding the quality and appropriateness of resources provided and thereby aid in improving the value of healthcare delivery. Previous studies already showed considerable geographical variation in healthcare at the EOL. However, the studies that did so are mostly from the USA [8,15,16] and focus mainly on hospital care [17–20]. As avoidable hospitalizations are seen as an indirect measure of access to effective primary care [21], we hypothesized that receiving primary and long-term care can avoid hospital admissions or that primary and long-term care at home in some way substitutes for variation in hospital care utilization. We therefore believe that investigating the complete cycle of care is important and will fill a gap in the literature.

Moreover, previous literature mainly addressed variation for a single time period [22,23] and only analyzed claims data on a diagnosis-related-group (DRG) level. Using national data for the period 2013–2015, this study will not only assess the geographic variations in hospital care on a detailed level for multiple years for Dutch LC patients but will also examine the degree to which the use of these services is associated with out-of-hospital services within geographic regions.

## Methods

### Study design and data sources

This cross-sectional study analyzed regional medical practice variation of EOL care during a patient’s last 6 months of life across 90 regions defined by their two-digit residential postal code as opposed to where the service is provided. This corrects for the ‘border crossing’ among geographic areas seen as a result of the existence of a referral center distant from the patient’s place of residence [24]. If more than one address was registered during the EOL phase, the postal code for the region of residence at the time of death was used.

Two data sources were used in this study. The first one is a Dutch database of administrative hospital data of all Dutch-insured patients (99% of Dutch population). In the Netherlands, hospital care reimbursements are based upon DRGs. These packages of care entail an average of all healthcare costs for a combination of various treatments; however, they do not give insight into the actual care provided. This database included both DRGs as well as the healthcare activities before they were grouped into DRGs. This meant we could analyze the actual care provided.

Healthcare activities are registered by all Dutch hospitals (8 academic and 82 general hospitals) and by more than 300 independent treatment centers. The data also contain information regarding the treating specialist and diagnosis (ICD-10 codes were unavailable), the institution providing care, 4-digit postal codes and the age and gender of patients. For 2013 and 2014, the data covered 95% of the delivered and billable care in these years in the Netherlands. For the care registered in 2015, this was approximately 70%, due to administrative delays in registries at the time the present analyses were carried out.

The second database comprises hospital care on a DRG level and information regarding the treating specialist and diagnosis and primary and long-term care claims data for all Dutch-insured persons. Both databases had pseudonymized patient IDs but could not be linked because these IDs were different in both datasets.

Information regarding age and gender were available in both datasets. Additionally, data on socioeconomic status (SES) were retrieved from the Social and Cultural Planning Office (SCP) and linked by four digital postal codes to our data. SCP calculated SES scores based on information regarding education, income and position in the labor market. The scores indicate the social status of a postal code region, relative to other regions in the Netherlands and cannot be interpreted purely as a number.

### Study population and selection

We included Dutch inhabitants who died in 2013, 2014 or 2015 with the diagnosis of LC. In the Netherlands, each DRG is claimed under a specialty and a diagnosis code. The medical advisors of the National healthcare Institute of the Netherlands provided us with the codes specific for LC patients. With these codes, for both datasets, we included patients if there were claims registered under specialty ‘lung diseases’, diagnoses ‘Tumors NSCLC’, ‘Tumors SCLC’; specialty ‘surgery’, diagnosis ‘neoplasm bronchus lung’ and specialty ‘internal medicine’, diagnoses ‘small and nonsmall-cell bronchus carcinoma’. Patients were excluded if they were younger than 18 years.

### Outcomes

Together with experts in the field of oncology, internal medicine and palliative care, and based on the literature addressing costly and potentially aggressive EOL treatments, we included the following hospital services for analyses: hospital admission days, emergency room (ER) contacts, chemotherapy, radiotherapy, computed tomography (CT) scans and intensive care unit (ICU) admissions [20,25–27]. Due to the cost saving and quality improving evidence for palliative care consultations, they were also selected. Secondly, hospital admission days and ER contacts were correlated with GP care and long-term home nursing care and assistance for daily life activities (ADL).

### Analysis of geographic variation in medical practice

Variations in medical practice were analyzed with the formula:

(1) \begin{eqnarray*} &&\hskip-12pt\mathrm{Regional}\ \mathrm{medical}\ \mathrm{practice}\ \mathrm{variation}\ \mathrm{score}\nonumber\\ &&\hskip-12pt \quad=\,\frac{\mathrm{Observed}\ \mathrm{regional}\ \mathrm{volume}}{\mathrm{Expected}\ \mathrm{regional}\ \mathrm{volume}}\ast \mathrm{National}\ \mathrm{average} \end{eqnarray*}

The ‘expected regional volume’ was determined with a logistic regression analysis adjusting for age, sex and SES [28,29] for each outcome to account for differences in patient characteristics between regions and years. Specific cut-off points for each outcome in the logistic regression were determined and were based on the national average number of treatments per person in the last 6 months of life, for patients receiving this specific treatment. The clinical relevance of these cut-off values was verified with the experts and the rationale behind this was that if someone receives more than the average number of treatments, then the healthcare utilization was indicated as high. The outcomes for the different hospital services were ten or more hospital admission days, one or more ER-contact, two or more chemotherapy treatments, two or more radiotherapy fractions and two or more CT-scans during the last 6 months of life.

The regression analyses resulted in estimated probabilities per patient. For each region, all estimated probabilities for the patients living there were summed up in order to obtain the expected volumes for each region. Next, the expected volume of each region was divided by the observed volume in that region and multiplied by the national average to obtain a regional medical practice variation score and standardized to 1000 patients. Regions were only included in these calculations if there were 30 or more deceased patients with LC in this region. This score can be interpreted as the age, sex and SES adjusted number of patients with the specific outcome per 1000 deceased patients.

We calculated two measures of geographic variation: the 95/5^th^ percentile ratio (factor score, Textbox 1) and the 95/5th coefficient of variation (CV95/5) [30]. Finally, an average factor score for the 3 years was calculated. We could only calculate factor scores for treatments that were registered in at least 95% of the regions as dividing by 0 would lead to an infinite number. This means that we were not able display results for palliative care consultations, ICU admissions (2013, 2014 and 2015) and radiotherapy (2015).

Textbox 1 Example factor score calculation.

The factor score (FS) is a measure to depict the degree of variation between hospitals based on the medical practice variation scores. It shows by which factor the highest score differs from the lowest score. We present the factor score based on the 95^th^ and 5^th^ percentile of the distribution of the medical practice variation scores. A factor score of 2 means that the patient visiting the hospital in the 95% percentile has a 2 times higher chance of receiving the treatment as opposed to him/her being admitted to the hospital in the 5% percentile.

*Example*: If for clinical admissions, the P5 score is 361 and the P95 score is 655, then, $FS=\frac{655}{361}$.

After this, for each region, we calculated the mean number of GP consultations, GP home visits and visits of a long-term home nurse for both home nursing activities and ADL per deceased LC patient. Using Spearman Rank order test, we assessed the correlation between these and the adjusted regional medical practice variation scores for hospital admission days and ER-contacts. To assess the robustness and consistency of the results, we stratified all analyses by year.

Analyses were performed with SAS Enterprise Guide 9.2.

## Results

### Patient characteristics

Overall, there were more men than women (60.0%, Table 1). We identified more patients in the primary care database. Mean age at death varied between the two databases. The patient characteristics did not differ between the years.

**Table 1:** Characteristics of all deceased patients with lung cancer in 2013–2015 by database

		Hospital care database	Primary and long-term care database
N		25 533	31 698
Gender (%)	Male	15 324 (60.0)	19 038 (60.1)
	Female	10 209 (40.0)	12 660 (39.9)
Mean age at death	Male	70.6	71.1
	Female	67.1	68.0

### Regional medical practice variation

The degree of variation is presented in Table 2. All investigated hospital services vary by two-fold or more. With a mean factor score of 3.6, the largest variation over the 3 years is found for two or more radiotherapy fractions. Note, with 335.5, the absolute difference was largest for ER-contacts. The factor scores of 2015 are higher compared to the scores in 2013 and 2014.

**Table 2:** Degree of regional variation in use of hospital care across two-digit postal code regions for Dutch lung cancer patients, described by factor scores and absolute difference of the P95-P5-adjusted regional medical practice variation scores

Treatment	2013			2014			2015		
	Mean 3 year factor score	Medical practice variation score (range)	Factor score	CV95/5	Medical practice variation score (range)	Factor score	CV95/5	Medical practice variation score (range)	Factor score	CV95/5
Radiotherapy fractions (2+)	3.6	86.8–413.5	3.2	0.25	91.2–379.4	4.0	0.20	^1^	^1^	0.51
Chemotherapy (2+)	2.6	153.6–413.3	2.0	0.15	155.5–450.0	2.9	0.17	107.4–391.6	2.9	0.20
CT-scans (2+)	2.6	153.3–409.1	2.1	0.15	155.5–447.5	2.9	0.17	107.5–391.7	2.9	0.20
ER-contacts (1+)	2.3	234.9–707.7	1.6	0.16	394.9–707.8	1.7	0.12	183.7–659.8	3.6	0.23
Hospital admission days (10+)	2.5	209.9–526.9	2.0	0.15	219.6–519.1	2.4	0.15	144.4–449.8	3.1	0.20

^1^No factor scores calculated due to > 5% regions with score of zero. CT-scan, computer tomography scan; CV, coefficient of variation; ER-contact, emergency room contact

### Substitution of hospital care by GP and long-term home care

Table 3 presents the results regarding the hypothesized substitution effect for out-of-hospital services. All correlation coefficients were weak, and most were nonsignificant. Only the adjusted regional medical practice variation scores for hospital admission days and ER days were statistically significantly correlated with the mean number of GP visits (*P*-value: < 0.001, 0.01, respectively) in 2014. However, the strengths of these correlation coefficients were very low (ρ: 0.39, 0.30 respectively).

**Table 3:** Spearman rank correlation between adjusted regional medical practice variation scores for ER and hospital admissions and the mean regional primary and long-term care utilization

Year	Adjusted medical practice variation score for		Mean number of GP home visits	Mean number of GP consulta-tions	Mean number of home nursing hours^1^	Mean number of ADL hours^1^
**2013** (*N* = 85)	Hospital admission days	Correlation coefficient	−0.13	0.09	0.05	−0.05
		Sig. (2-tailed)	0.24	0.41	0.64	0.63
	ER-contacts	Correlation coefficient	−0.04	0.05	0.06	0.03
		Sig. (2-tailed)	0.69	0.66	0.61	0.81
**2014** (*N* = 79)	Hospital admission days	Correlation coefficient	**−0.39**	−0.02	−0.07	−0.07
		Sig. (2-tailed)	<0.001	0.88	0.52	0.54
	ER-contacts	Correlation coefficient	**−0.30**	−0.07	−0.06	−0.04
		Sig. (2-tailed)	0.01	0.56	0.62	0.71
**2015** (*N* = 85)	Hospital admission days	Correlation coefficient	−0.14	−0.10	−0.10	0.07
		Sig. (2-tailed)	0.22	0.39	0.36	0.53
	ER-contacts	Correlation coefficient	0.09	−0.02	0.07	0.11
		Sig. (2-tailed)	0.40	0.84	0.54	0.31

^1^Provided under the long-term care act. ADL, assistance daily life; GP, general practitioner; ER-contact, emergency room contact.

## Discussion

This study has three main findings. First, our results show that regional medical practice varies during the last 6 months of life for hospital care in patients with LC in the Netherlands, by at least twofold. Thus, the place where patients live is associated with the type and intensity of care they receive. Second, the presence of variation is persistent over the course of 3 years and seems to increase. Third, the degree of variation of hospital services was not (strongly) associated with the degree of healthcare utilization outside the hospital in terms of GP and long-term care (substitution).

A study by the OECD, 2014 also found hospital medical admissions to vary by two-fold or more within countries which they noted as considerable [31]. Furthermore, Goodman et al. showed that, during the last month of life, the number of hospital days varied with a factor 2.7 between regions in the United States. However, he found that life-sustaining treatments varied more than fivefold among patients being treated in academic medical centers [32], which is much higher compared to our findings. However, Goodman investigated cancer patients in general and not a more specific group like LC patients.

Multiple factors are important when explaining the variation. For instance, we know that the EOL care can be supply-sensitive [33]. Living near a specialized oncology treatment center or academic treatment center might lead to different care as opposed to living near a small general hospital [34]. Also, the local bed supply, rather than patient preferences, have been shown to explain the differences in EOL care among patients [35]. Studies have also shown that physicians adapt their admission and discharge decisions to the availability of intensive care unit beds, admitting more patients with less severe illness and extending the length of stay when more beds are available [36]. Also, patient preferences could influence the care provided or utilized. For instance, religion is associated with receiving intensive life-prolonging EOL care [37]. Lastly, differences in speed and uptake of medical and organizational innovations and thus access to EOL care might also explain part of the (probably temporal) variation we found [38]. The fact that a small country like the Netherlands has over 60 palliative care networks, which are all organized differently may illustrate this.

It is known that patients have different preferences regarding treatment in the last phase of life. However, it is also known that generally, the healthcare provided at the EOL does not always reflect these preferences [1,39]. Our data did not permit a study of patients’ preferences.

The question remains whether the variation we found is unwarranted. Even though our results cannot answer this, previous research suggests that a lot of admissions in oncology patients are due to acute cancer-related conditions such as pain and fever and are therefore potentially avoidable [40,41]. The large degree of variation in ER-contacts and hospital admissions indicates that some of the patients underwent a prolonged treatment which probably led to unwanted admissions through cancer-related complications in some cases. Also, the appropriateness of chemotherapy near the EOL is complex. There is an ongoing debate regarding the benefits of chemotherapy and other life-sustaining procedures in these patients [42] and the question is raised whether the high number of patients with two or more chemotherapy treatments found in some regions is warranted. Unfortunately, we were unable to determine whether the chemotherapy provided was life-prolonging or given to treat symptoms and pain and in line with the patient’s wishes.

### Strengths and limitations

A considerable strength of our study is the analysis of data with a national coverage at the level of healthcare activities. The advantage of this is that the healthcare registered can be analyzed in greater detail than most published studies did. A second strength is that the data of the entire chain of healthcare made it possible to study trade-off effects between variations in hospital and outpatient care. Finally, we chose to present factor scores based on the P95-P5 range. By doing so, we showed that, even when we exclude outliers, variation is still substantial. Additionally, by performing the analyses over several years, we have shown that the variation we see is persistent. However, the 70% coverage of all billable claims in 2015 may be a contributor to the deviating factor scores for this year compared to the two previous ones.

We adjusted for age and sex to remove the effects of differences in populations. This removes part of the variation explained by comorbidities, especially the age-dependent ones. However, not completely, it does not adjust for other, possibly important factors. For instance, cancer staging is likely to be associated with the treatment plan of the patients and the care that is provided. National data show that this varies little over the investigated regions. Therefore, we believe this to have little to no effect on our results. We also adjusted for SES on an aggregated level; individual SES would be a better predictor of utilization of EOL treatments. Lastly, we were unable to take patient-preferences or the supply of care into account.

Care in the primary/long-term care setting was not utilized sufficiently by the required number of LC patients to perform medical practice variation analyses. Thus, we correlated regional medical practice variation scores in secondary care with absolute numbers of primary and long-term care. This meant that we compared adjusted utilization to crude utilization.

A final limitation is the fact that we were only able to analyze the variation on a regional level, based on where a patient lived and not on an institutional level. The drawbacks are that the residential area does not necessarily match with the treatment seeking area, and regions are difficult entities to hold accountable for delivered care.

### Recommendations

More research is necessary to identify the underlying reasons causing variation and to determine which part of it is unwarranted.

Our suggestion for the next step would be to identify and characterize hospitals or hospital regions with high and low scores. This would also overcome the issue that the residential area does not necessarily match the area where treatments are provided. Next, it is important to further investigate possible factors such as the supply of care and patients’ and doctors’ preferences influencing the care that is given at the EOL. Finally, assessing whether a reduction in variation increases patient outcomes would give more insight into the warrantedness of the present variation. Medical specialists should be aware of the difficult decision-making that patients with cancer face near the EOL, how different their perspective is about treatment benefits [43] and how their preferences vary especially because we know that a palliative treatment aim could prevent hospital admissions [44]. Timely palliative care (advance care planning) and shared decision-making can play a role in this [45–47]. Ultimately, this will provide patients and professionals insight into the drivers of variation and tools to improve EOL care.

## Author’s Note

The lead author affirms that the manuscript is an honest, accurate and transparent account of the study being reported, that no important aspects of the study have been omitted and that any discrepancies from the study as planned (and, if relevant, registered) have been explained.

## Funding

This work was supported by the National Health Care Institute of the Netherlands. The funding organization did not participate in the design and conduct of the study; analysis, and interpretation of the data; preparation, review, or approval of the manuscript; and decision to submit the manuscript for publication. They did participate in collection and management of the data as they are the processors of the dataset. All researchers are independent of the funding party.
